# Advanced mucinous colorectal carcinoma in a 14-year old male child: A case report and review of the literature

**DOI:** 10.1016/j.ijscr.2020.04.030

**Published:** 2020-05-11

**Authors:** Alex Mremi, James J. Yahaya

**Affiliations:** a Department of Pathology, Kilimanjaro Christian Medical Center (KCMC), Moshi, Tanzania; b Department of Biomedical Science, College of Health Science (CHS), The University of Dodoma, P. O. Box 395, Dodoma, Tanzania

**Keywords:** Mucinous colorectal cancer, Child, Poor prognosis

## Abstract

**Introduction:**

ColorectaI carcinoma is extremely rare in children and presents with a poor prognosis.

**Presentation of case:**

The present report describes the case of a 14-year old male child who presented with complaints of general weakness and recurrent abdominal pain, caused by a mucinous adenocarcinoma of the transverse colon (Dukes stage which was inoperable.

**Discussion:**

Mucinous histopathological type is the most common type with increased ability to invade the adjacent stromal tissue. This biological behaviour has been reported to be attributable to its aggressive behaviour. Additionally, delay of diagnosis of colorectal carcinoma in children accounts for the advanced disease at diagnosis.

**Conclusion:**

Colorectal carcinoma occurring in both children and adolescents usually have poor prognosis because of not having specific symptoms which contributes to delay of diagnosis. Mimicking of its symptoms with other non-malignant conditions such as intestinal obstruction and acute appendicitis also has been found to contribute to delay of diagnosis as it was in our present case.

## Highlights

• Colorectal carcinoma in children is extremely rare and it carries poor prognosis.
• Diagnosis of this malignancy in children usually is done at advanced stages.
• Mucinous variant is the most common histological type of colorectal cancer in children.
• Screening for colorectal cancer in children helps to diagnose the disease at early stage.

## 1 Introduction

Colon cancer in children and young adults has been reported to be not common albeit of a few cases that have been reported. It has been estimated that 1–2 children and/or adolescents are diagnosed with colon cancer in 1 million people globally; however, the incidence seems to be increasing [1]. The age-adjusted incidence rate of colon cancer in the population of adults is said to be 43.7 people per 1, 000, 000 [2]. Over 70% of colon cancer cases are sporadic and about 20% are familial [3]. The familial colon cancer in children and adolescents is closely linked with the inheritance of familial syndromes such as familial adenomatous polysis, juvenile polyposis, Lynch syndrome, hereditary nonpolyposis colon cancer (HNPCC), inflammatory bowel diseases (IBDs), neurofibromatosis type 1 (NF-1) among many others [3,4].

The presentation of colon cancer in the pediatric population is similar to that of the general population [5]. Abdominal pain, constipation, body wasting, melena and anaemia among others, have been reported to be the common presenting features of colon cancer in both children and adults. Abdominal pain seems to be not indicative of colon cancer symptom in the pediatric population due to the fact that, there are other conditions including intestinal obstruction and/or appendicitis which are more likely to present with abdominal pain, hence leading to delay of diagnosis in children and adolescents because of them being more common in the pediatric population than in adults [6,7].

This paper reports a case of a 14-year old male child who was diagnosed with a stage IV (T4bN0M1) mucinous variant of colon cancer which was poorly differentiated and was involving the right side of the transverse colon with mild anaemia which makes this case novel. This patient comes from a family with unknown any cancer history due to lack of cancer screening in the country and also financial constraints from the family to perform cancer screening. This case will help to add insightful information to the clinicians handling children particularly in areas with possibility of helminthic infestations which is actually more likely to cause delay of diagnosis for colon cancer as it was the case for our patient. The work has been reported in line with the SCARE criteria [8].

## 2 Presentation of a case

A 14-year old male was referred from a health center to our medical center with a chief complaint of long standing severe abdominal pain and tenderness especially in the right side flank region associated with vomiting after meals. The patient had five days history of vomiting which was projectile and fluidy in nature. It was reported that, while the patient was at the health center, he was treated with antibiotics (metronidazole and ciprofloxacylin) and analgesics. The patient also has had laparotomy done at the health center two months before being referred to our medical center as it was thought that the patient had acute appendicitis. The patient remained at home for about three months before being brought at our medical center as the diagnosis was initially missed at the health center.

On physical examination, the patient was ill looking, dehydrated, non-jaundiced, and afebrile with abdominal pain which was more severe in the right side and it was radiating to the umbilicus region with mild anaemia. The pain was sharp in nature without relieving factors. Failure to pass stool was also reported. Other systems were normal. The patient had distended abdomen with engorged superficial abdominal blood vessels. Tenderness on palpation mostly in the epigastrium region, dullness on percussion, rebound tenderness was also present. Bowel sounds were exaggerated (very audible). There was a surgical scar on the midline part of the abdomen. The body temperature was 37 °C, Total WBC = 7.6 × 10^6^/μl (normal), haemoglobin level =11.5 g/dl (mild anaemia), platelet = 386 × 10^3^/μl (normal), neutrophile = 7.86 × 10^3^/ μl (normal), creatinine =137.4 mmol/l (normal), potassium =2.75 mmol/l (low), sodium =122.64 mmol/l. Intravenous (IV) normal saline (ringer lactate) solution of 1.5 L per 24 h, IV centriaxine 1 gm stat, IV metronidazole 500 mg stat were given. Digital rectal examination revealed smooth and free rectal mucosa with normal sphinter’s tone. The anal verge was also normal and the examination glove was not stained with blood. The clinical diagnosis was intestinal obstruction. The patient was admitted in the pediatric ward.

Laparotomy was one by a general surgeon with an assistance of a resident. Under sterile condition, in a supine position, the patient was cleaned and draped through then a midline an incision was made to open the abdomen. Upon opening the abdomen, it was found that, the appendix was ruptured and they attempted to drain the pus but the large bowels were massively adhered to the peritoneum. The omentum was adhered to the ileocaecum and the caecum was adhered to peritoneum. Also there were matted bowel loops towards the mid transverse colon and splenic flexture where two firm tumours were seen after releasing adhesions. A rectal tumour was also seen with frozen pelvis. The entire bowels, mesentery, stomach and liver were noted to have tumour seedlings as well as anterior abdominal wall.

Clinical stage cT4bNoMo was given. The large colon was grossly distended. Release of the matted bowels, loop colostomy, bowels decompression and an incisional biopsy was taken from the transverse colon because the tumour was inoperable. Abdominal irrigation with normal saline, insertion of a drainer and closure of the abdomen in layers was done. Cleaning of the abdomen using metronidazole was done followed by closure of the abdomen.

**Fig. 1:**
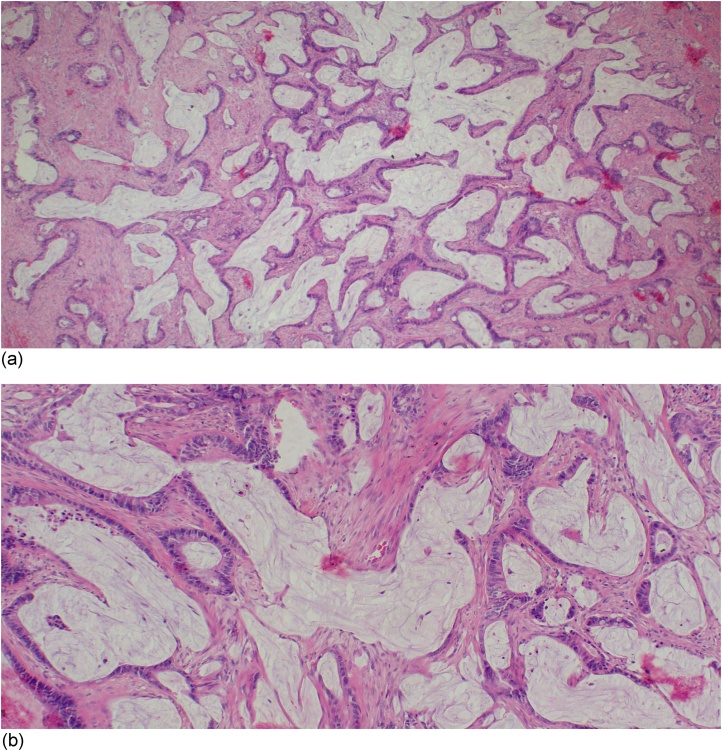
(a) Islands of mucinous lakes infiltrating the stromal tissue (H&E stain, x40). (b) The infiltrating lakes of mucin and infiltrating tumour glands are also evident (H&E stain, x100).

Macroscpically, the submitted piece of tissue was gray-white, firm in consistency and it measured 6 × 3 x 2 cm. Microscopically, the tissue was infiltrated by a tumour which was richly composed of islands of mucinous lakes (Fig. 1a) and in other areas malignant glands (Fig. 1b) were evident. The tumour had involved the subserosal layer. The tumour was confirmed to be mucinous colorectal adenocarcinoma with Dukes stage D. It was unfortunate that, the chest and abdominal survey for metastasis was not done due to the fact the parents could not afford to pay for the computed tomography scan. This limited the patient to be evaluated for possible distant organ involvement by the primary tumour.

After one week after operation the patient was discharged home on antibiotics (Metronidazole 500 mg IV tds, Cefriaxone 1 gm stat IV) and pethidine 36 mg IM as palliation management because it was not possible for the patient to start chemotherapy because of being unstable. Two weeks later when the parents were contacted for requesting the patient to be taken for clinical evaluation, it came to be known that, the patient had died two days before the parents were contacted.

## 3 Discussion

Our patient initially was not thought to have colon cancer due to having haemoglobin level of 11.5 g/dl. The presentation of severe abdominal pain although the pain was more to the right flank region, still the child was thought to have intestinal obstruction based on the place of residence reported to have significant *Ascariasis lumbricoides* infestation in the country.

Experience has shown that, the presenting signs and symptoms for colorectal cancer are usually not specific and they may include mild abdominal pain, constipation, diarrhea, hematochezia, weight loss and anaemia and have been found to be underestimated. Also, it has been found that CRC presents clinically in a similar way in both children and adults and the median duration for patients to present with symptoms of CRC is approximately three months [9]. Therefore, emphasis must be made to provide education to health providers particularly those who are working at the low health facilities for them to have high index of suspicion for timely diagnosis of this cancer in the pediatric population [10]. Ascending and transverse colon cancers are usually diagnosed late because they usually present with symptoms of intestinal obstruction unlike frank bleeding which marks substantially advanced tumour. This is why it took almost three months for the confirmatory diagnosis of mucinous colon cancer for our case to be obtained because initially it was thought that the patient had intestinal obstruction.

Regarding anatomical distribution of CRC along the colon, studies have shown that, in adults CRC occurs more common to the left side whereas in children the site of involvement is varying and has been found to be equally distributed in all parts of the colon [11]. The rarity of this cancer in this population could also explain the lack of studies involving large sample size. In one study by Ashley et al [5] which is thought to be one of the studies with large sample size (77 cases) for colon cancer in the pediatric population reported that, 76% of the cases were clinically presenting with abdominal pain, constipation, wasting and anaemia [3,5]. Presentation of mild anaemia (Hb level of 11.5 g/dl) in the present case makes the case novel because majority of cases with colon cancer have been reported to present clinically with severe anaemia. In one study done by Edina et al [12] which was determining prevalence of anaemia in patients with colon cancer, it was reported that 74.7% of the patients had anaemia. In the pediatric population, CRC occurs relatively more frequently in males than in female children with ratio of 2:1 [13].

To diagnose CRC, mainly requires a high index of suspicion which can be confirmed by sigmoidoscopy or colonoscopy and biopsy [13]. The use of imaging diagnostic tests such as CT and positron emission tomography (PET) has been found to assist in detecting the cancer [13]. The genetic screening of family members should be done to rule out the various hereditary polyposis syndromes. Additionally, endoscopy, abdominal ultrasound and x-ray and barium enema are also be used to detect presence of the tumour along the colon [7]. Screening can also result in CRC detection and diagnosis at an early, localized stage when the relative five-year survival rate for patients is 90% [14]. Ashley et al reported that, 50% of children and adolescents who were included in the study had microsatellite instability [5].

The mainstay of treatment of CRC in both children and adults is surgery which depends on extent of extension of the disease. Complete tumour resection including dissection of retroperineal lymph nodes is done in patients with resectable tumour and it has been found to have the greatest impact on the overall survival of the patients [13]. For cases with advanced tumour stages at presentation (stage III and IV), surgery becomes challenging and sometimes not possible [5]. The role of chemotherapy in paediatric CRC is controversial [9]. In adults, the FOLinic acid, 5-Fluorouracil and OXaliplatin (FOLFLOX) regimen of chemotherapy has been reported to demonstrate a clear benefit in patients with stage III and IV disease and is actively evolving also [14]. Radiotherapy is usually advocated when CRC involves the rectum. The dose varies and may be provided after or before chemotherapy [5].

The prognosis of CRC in children and adolescents is poorer than in adults. A number of prognostic factors have been reported to be associated with the poor clinical outcomes of CRC in children and adolescents. One of the reasons for the poor prognosis is delay in diagnosis of CRC in children compared to adults in whom colonoscopy would promptly help to detect the disease at early stage. It has been reported that 60%–80% of CRC in children and adolescents is detected at advanced stages (stage 3 and 4) [1]. This is due to the reason that, the signs and symptoms of CRC in children tend to be vague and unspecific almost mimicking other commonest conditions including intestinal obstruction and appendicitis [10].

Another reason major reason is that, most of children and adolescents are diagnosed with mucinous and/or signet ring cell variants of CRC which are known to carry poor prognosis. For example, in one of the series involving 77 of children and adolescents with CRC, it was found that 62% and 48% of the patients had mucinous and signet ring cell variants of CRC [5]. Further findings of this study reported that patients with non-mucinous CRC had better prognosis than those with mucinous CRC (p = 0.02). on the other hand, patients with greater than 10% proportion of signet ring cells they had also poorer prognosis than the ones with no signet ring cells or less than 10% of signet ring cells (p = 0.04) [5]. Moreover, adults commonly have tubular variant of CRC which has good prognosis.

In another study which was involving 159 patients from the Surveillance, Epidemiology and End Results (SEER) data, it was found that, mucinous and signet ring cell variants of CRC had 5-year survival rate of 27% and 13%, respectively [15]. Fielding et al [16] reported that, increased carcino-embryonic antigen (CEA) tumour marker is associated with increased possibility of metastasis of CRC patients at presentation. Also in the same study it was reported that, DNA ploidy (DNA content of malignant cells nuclei) was used to determine prognosis of patients with CRC. Patients with high diploid DNA had better prognosis than with aneuploidy DNA state [16]. Other prognostic factors include tumour stage, tumour grade, lymph node metastasis, vascular and perineural invasions [17].

Survival of children and adolescents diagnosed with CRC is poor compared to that of adults. In the study done by Iyad et al, the 5-year and 10-year overall survival estimates for children and adolescents were 40% ± 4.2% and 31% ± 4.4%, respectively (median follow-up, 4.9 years [95% CI = 2.3–7.0 years) whereas that of adults was 60% ± 0.10% and 54% ± 0.1% for the 5-year and 10-year overall survival, respectively (P < .001) [15]. This finding clearly shows the existing difference of prognosis for the two populations.

## 4 Conclusion

The delay in diagnosis of CRC in children as it was the case in our patient is of concern when it comes to improving prognosis of children with CRC. Lack of clinical features that are potentially linked to the presence of this malignancy is a major reason for the poor clinical outcomes. Therefore, there is a need for screening of the children for CRC particularly those with relatives having a history of CRC. Additionally, clinicians should always ensure that, any child in whom there are traces of signs and/or symptoms of CRC is vigorously examined and followed up in order to detect and treat the disease while is still at early stage.

## Declaration of Competing Interest

Authors declare that there are no conflicts of interest regarding this work to be disclosed.

## Sources of funding

This paper did not receive any specific grant from funding agencies in the public, commercial, or not-for-profit sectors.

## Ethical approval

Ethical clearance was obtained from the Research Ethics and Review Committee (CRERC) of the Kilimanjaro Christian Medical Center (KCMC) with a reference number CR-075/CRERC-01/20.

## Consent

Written informed consent to publish the information of the patient and images was obtained from the parents of the patient and it has kept for reference by the Editor-in-Chief of the journal when required.

## Author contribution

**James J Yahaya**: Performed conception, designing and drafting of the article. He also revised the manuscript critically for important intellectual content, and finally he provided approval of the version to be submitted. **Alex Mremi**: Performed acquisition of data, designing and revising the manuscript critically for important intellectual content, and finally he approved the version to be submitted.

## Registration of research studies

1 Name of the registry:
2 Unique Identifying number or registration ID:
3 Hyperlink to your specific registration (must be publicly accessible and will be checked).

## Guarantor

Dr. James J Yahaya.

## Provenance and peer review

Not commissioned, externally peer-reviewed.
